# The Use of Insecticides to Manage the Western Corn Rootworm, *Diabrotica virgifera virgifera*, LeConte: History, Field-Evolved Resistance, and Associated Mechanisms

**DOI:** 10.3390/insects12020112

**Published:** 2021-01-28

**Authors:** Lance J. Meinke, Dariane Souza, Blair D. Siegfried

**Affiliations:** 1 Department of Entomology, University of Nebraska, Lincoln, NE 68583, USA; 2 Entomology and Nematology Department, University of Florida, Gainesville, FL 32611, USA; dariane.souza@ufl.edu (D.S.); bsiegfried1@ufl.edu (B.D.S.)

**Keywords:** chemical control, pest management, insecticide metabolism, *Diabrotica virgifera virgifera*, insecticide resistance

## Abstract

**Simple Summary:**

The structure of agricultural enterprises in the western United States Corn Belt (large irrigated monocultures, continuous planting of maize, strong aerial pesticide application and livestock industries) has led to a tradition of extensive insecticide use over time to manage the western corn rootworm, *Diabrotica virgifera virgifera* LeConte (Dvv) a key insect pest of maize. Dvv damages maize roots, which can cause maize plant instability, reduced plant growth, and significant yield loss. Long-term insecticide use has contributed to Dvv becoming resistant to cyclodiene, organophosphate, carbamate, and pyrethroid insecticides since the 1950s. This paper reviews the historical and current use of insecticides in Dvv management programs and Dvv adaptation to insecticide use. Currently, insecticides have a reduced role in Dvv management programs but are still used as complementary tactics with other management approaches. Past history suggests that the probability of selecting for resistance to any future Dvv control technology will be high if it is not used within an integrated pest management framework with other tactics.

**Abstract:**

The western corn rootworm, *Diabrotica virgifera virgifera* LeConte (Dvv) is a significant insect pest of maize in the United States (U.S.). This paper reviews the history of insecticide use in Dvv management programs, Dvv adaptation to insecticides, i.e., field-evolved resistance and associated mechanisms of resistance, plus the current role of insecticides in the transgenic era. In the western U.S. Corn Belt where continuous maize is commonly grown in large irrigated monocultures, broadcast-applied soil or foliar insecticides have been extensively used over time to manage annual densities of Dvv and other secondary insect pests. This has contributed to the sequential occurrence of Dvv resistance evolution to cyclodiene, organophosphate, carbamate, and pyrethroid insecticides since the 1950s. Mechanisms of resistance are complex, but both oxidative and hydrolytic metabolism contribute to organophosphate, carbamate, and pyrethroid resistance facilitating cross-resistance between insecticide classes. History shows that Dvv insecticide resistance can evolve quickly and may persist in field populations even in the absence of selection. This suggests minimal fitness costs associated with Dvv resistance. In the transgenic era, insecticides function primarily as complementary tools with other Dvv management tactics to manage annual Dvv densities/crop injury and resistance over time.

## 1. Introduction

**Figure 1:**
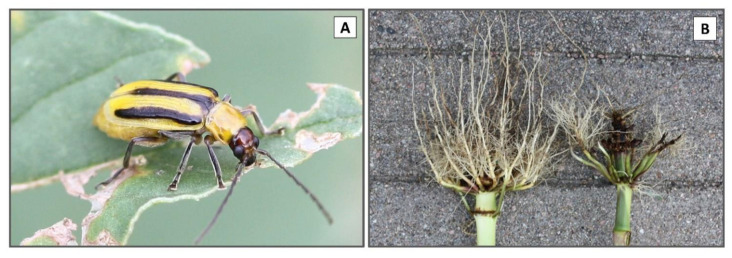
(**A**) Adult *Diabrotica virgifera virgifera* LeConte; (**B**) example of severe root injury from *Diabrotica virgifera virgifera* LeConte (Dvv) larval feeding that can occur when Dvv larval density is high (right) versus uninjured root (left); photos by L. J. Meinke.

The western corn rootworm, *Diabrotica virgifera virgifera* LeConte (Dvv) is a galerucerine Chrysomelid beetle (Figure 1A) that is one of the most significant insect pests of maize (*Zea mays* L.) in the United States (U.S.). Annually, this species is responsible for over $1 billion in control costs and yield losses [1,2]. Similar to other *Diabrotica* species, the larvae are root feeders and adults feed on above-ground plant tissues [3,4]. Dvv larvae survive only on a small number of grass species [3,4,5,6,7,8] while adults feed primarily on pollen and reproductive tissues of a variety of plants [9,10]. Maize is the primary Dvv host in modern agroecosystems [10,11] and is strongly attractive to wild-type adult Dvv [12,13,14]. The initial Dvv species description was made in 1868 from collections made in what today is Wallace Co., KS [15] but phylogenetic research points toward a species origin in Mexico or Central America [16,17]. It has been hypothesized that Dvv may have survived at low densities on native grasses such as western wheatgrass, *Pascopyrum smithii* (Rydb.) at the time of initial discovery [7]. The pheromone of Dvv is very efficient at ultra-low levels, which supports the low Dvv density hypothesis before adaptation to maize monocultures [11]. It is unclear when Dvv arrived in what today is the southwestern U.S. but records document that Dvv did not occur east of western Kansas, Colorado, and southwestern Nebraska, U.S. prior to the 1920s [18].

Dvv was first recorded feeding on maize roots at Ft. Collins, Co. in 1909 [19]. Annual rotation from maize to a crop that would not support Dvv larval survival was the recommended Dvv management tactic as early as 1930 in southwestern Nebraska [20,21] but the profitability of maize led some growers to start planting continuous maize (maize planted for ≥2 years in one location). As agriculture developed in western areas of Nebraska during the 1930s sporadic reports of larval Dvv injury were reported in continuous maize [20,21,22]. By the 1940s, Dvv injury to continuous maize (Figure 1B) in central Nebraska was common and was facilitated in part by the introduction of irrigation systems and synthetic fertilizer [23,24,25,26]. This agricultural system became very profitable and helped meet the demand for maize from a growing confined livestock industry. Continuous maize provided optimal conditions for the build-up of Dvv densities, increasing larval injury and greater adult dispersal from infested fields [18]. The large monocultures of continuous maize may have been the bridge needed to jump-start the fairly rapid range expansion that progressed through the U.S. Corn Belt reaching NJ, USA by the 1980’s [18,27].

**Figure 2:**
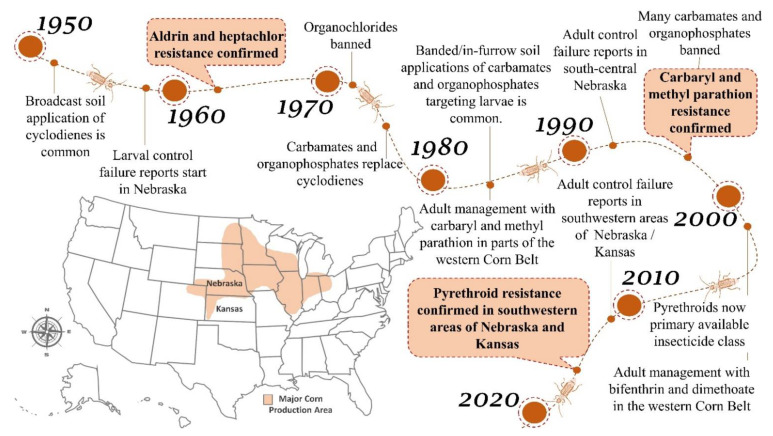
The agricultural region in the midwestern U.S. that is a major producer of maize is called the Corn Belt. This figure shows the major maize grain production area within this region, a Dvv insecticide use/field-evolved resistance timeline associated with continuous maize, and the geographic location of states Nebraska and Kansas where Dvv resistance to multiple insecticides has occurred. Maize grain production area is based on USDA-NASS 2015–2019 data [42,43].

The emergence of Dvv as a major insect pest coincided with the post-World War II emergence of agrochemical companies that synthesized and manufactured synthetic organic insecticides. The need for Dvv control in continuous maize created a niche that developed into a large insecticide market in the U.S. Corn Belt [28]. Evaluation of insecticide efficacy targeting larvae (soil insecticides) or adults (foliar applications) was a major focus of both industry and academia in the 1940s–1990s [25,29,30,31,32,33,34,35,36,37,38]. Seed treatments and transgenic plants that express proteins that are toxic to Dvv (plant incorporated protectants) were introduced and widely adopted in the 2000s, which reduced the role of insecticides as management tactics in continuous maize [26,39,40,41]. This paper reviews the history of insecticide use in Dvv management programs, Dvv adaption to insecticides (Figure 2), i.e., field-evolved resistance and associated mechanisms of resistance, plus the current role of insecticides in the transgenic era.

## 2. Western Corn Rootworm—Insecticide History

### 2.1. Soil Insecticides

The use of soil insecticides to manage Dvv in field maize was first demonstrated by Hill et al. (1948) [23] and Muma et al. (1949) [29] in Nebraska. Broadcast planting-time applications of the chlorinated hydrocarbons DDT and the gamma isomer of benzene hexachloride (BHC) were initially evaluated. BHC (lindane) significantly reduced Dvv larval densities, plant lodging caused by larval Dvv feeding, and in some trials, increased yield when compared to untreated maize. Soil application of DDT was found to be comparatively ineffective. Muma et al. (1949) [29] also reported that BHC persisted in the soil providing Dvv control the following season after initial application. In subsequent years, cyclodienes were shown to have activity against Dvv larvae [44] and along with BHC were recommended to growers for larval control (aldrin, chlordane: [45]; heptachlor: [46]). Large-scale broadcast soil applications of BHC and cyclodiene insecticides were commonly made to field maize during the 1950s. By 1959, almost 1000 metric tons of aldrin had been applied as a soil insecticide in Nebraska alone [47].

Ineffective control of Dvv larvae after application of a soil insecticide was initially observed in south-central Nebraska in 1959 [48]. The rapid development and continuance of the problem became more apparent by 1960–1961 [49,50,51]. In lab bioassays, LD50s of a Dvv population from a control failure area of south-central Nebraska were 78.8 and 43.2-fold greater for heptachlor and aldrin, respectively, than a population in eastern Nebraska where no larval control issues had been observed [52]. This was the first direct evidence of field-evolved resistance in Dvv to an insecticide. A follow-up study documented that susceptibility of Dvv to aldrin was highly correlated with previous insecticide use patterns in Nebraska. The LD50s of populations from high insecticide use areas around the Platte River Valley were up to 1000-fold greater than some populations from far western NE where little soil insecticide had been used [53].

Cyclodiene resistance in Dvv populations was limited to the western U.S. Corn Belt at this time because the eastern Dvv expansion across the U.S had only reached the Nebraska—Iowa border by 1954 [54] and western Wisconsin by 1964 [18]. Cyclodiene resistance spread throughout the existing Dvv range and was maintained in Dvv populations as the range expansion across the U.S. Corn Belt progressed. Populations along the expanding species boundary exhibited similar resistance levels to cyclodienes by 1964 [55] and high levels of resistance were present in the eastern U.S. during the 1980s even in areas where cyclodienes were not widely used before removal from the market by the U.S. Environmental Protection Agency (USEPA) in the 1970s [56]. Metcalf (1986) [57,58] noted that the rate of geographic spread was slower before than right after resistance evolved to cyclodiene insecticides. Metcalf inferred that a behavioral change associated with resistance may have led to increased movement of the range expansion front [57,58]. This hypothesis turned out to be unsupported as Dvv exhibited stratified dispersal with wide variation in rate of expansion depending on location and year [18].

Following widespread Dvv resistance to the environmentally persistent cyclodiene insecticides, there was a concentrated effort by state, federal, and industry scientists to discover and commercialize new insecticides with activity against Dvv [33]. Chlorinated hydrocarbon and cyclodiene insecticides were gradually removed from commercial use by regulation in the U.S. and replaced by organophosphate and carbamate insecticides [59]. The replacement insecticide classes were highly toxic, more expensive, and had shorter residual activity than the cyclodienes [33,59]. A shift from broadcast soil applications to applications banded over the row or placed in the seed furrow were adopted to reduce rates applied and subsequent cost [33]. Most soil-applied insecticides targeting larval Dvv are applied at planting-time, which often is 4–8 weeks before Dvv eggs begin to hatch. Therefore, historically, many products were granular formulations that would extend residual activity. Eventually, prophylactic use of soil-applied organophosphate or carbamate insecticides at planting became the primary Dvv management approach in continuous maize throughout the U.S. Corn Belt [28]. Prophylactic applications were often made without knowledge of the pest density present, which led to some unnecessary insecticide applications [60,61,62]. It was not until the 1980s that several insecticides in the pyrethroid insecticide class (bifenthrin, tefluthrin) were registered as Dvv soil insecticides in the U.S. [63,64].

During the 1970s to early 1990s, there were numerous studies conducted to understand environmental and agronomic factors that could impact soil insecticide efficacy and increase understanding of Dvv population dynamics when soil insecticides were used in continuous maize. Performance of insecticide applications of all classes can be inconsistent from year to year in the soil environment [59,65]. The response of an insecticide in soil is a complex interaction of the physicochemical properties of an insecticide, soil and environmental conditions, and the susceptibility of the target insect pest [65]. Very dry or excessively wet soils, high soil pH, or soils with high organic matter content have all contributed to variable soil insecticide efficacy when targeting Dvv larvae [28,66,67,68,69].

Organophosphate and carbamate insecticides are biodegraded by microbiological metabolism [59,70,71]. This is normally viewed as a positive characteristic since it prevents persistence of insecticides in the soil over time but can negatively impact soil insect control if biodegradation occurs rapidly and reduces insecticide residual in the soil [72]. Declining Dvv control with some carbamate or organophosphate soil insecticides was reported during the 1970s–1980s after consecutive years of application [59]. A key example was the carbamate carbofuran where studies documented poor Dvv control in soil plots treated with carbofuran at planting for successive years versus plots with no history of carbofuran use [73]. Subsequent research determined that rapid degradation of carbofuran by soil microbes in conditioned soils led to poor control and that field-evolved resistance to carbofuran in Dvv probably was not the causative factor [74,75]. A similar scenario occurred with the organophosphate isophenphos. This soil insecticide was initially marketed as Amaze^®^ in 1981 but by 1983, Dvv control failures were reported from various locations in the U.S. Corn Belt in fields where the product had been used the previous year [59]. Enhanced microbial biodegradation was found to be the cause of rapid loss of isophenphos in the soil [76,77] and the formulation was removed from the market soon thereafter [59]. In both of these examples, microbial degradation of the soil insecticide occurred between planting time and the Dvv egg hatch period reducing active ingredient in the soil.

Planting date and associated timing of insecticide application relative to Dvv population dynamics can also impact efficacy of soil-applied insecticides. Soil insecticides often perform better when planting date is later and insecticide application is closer to the occurrence of Dvv larvae in the field [78,79,80]. Before minimum or no-till practices were widely adopted, application of insecticides at cultivation during the Dvv egg hatch period was demonstrated as an alternative to planting-time applications especially when maize was planted very early in the season [81]. Reduced tillage practices do not appear to significantly impact Dvv control with soil insecticides [28,82,83].

The placement of soil-applied insecticides in a band over the row or placed in the seed furrow protects the main maize root system (reduces injury, lodging) but does not manage the Dvv population [28,83,84]. Little to no reduction in adult Dvv emergence from soil insecticide treated plots versus untreated plots is commonly reported [83,84,85,86]. This has been attributed to survival of Dvv larvae feeding on maize roots between rows that have grown outside the treated zone and in some cases differences in larval density-dependent mortality between treatments [83,86]. Therefore, there is often an inconsistent relationship between soil insecticide efficacy as measured by level of root injury and subsequent adult emergence. This built-in refuge is probably a major reason why field-evolved Dvv resistance attributed to direct selection of Dvv larvae has only been documented with broadcast-applied cyclodiene insecticides but not insecticides applied in-furrow or banded over the row [83,86,87].

The fitness of Dvv adults that emerge from soil insecticide treated fields is highly variable. Later mean adult emergence time, increased longevity, increased egg production, and variable sex ratios have been reported after Dvv larval exposure to organophosphate and carbamate soil insecticides [85,88,89,90,91], but results are inconsistent within products over years. Soil insecticide exposure does not appear to significantly affect egg viability [89,90]. Extended mean development time can also be caused by high Dvv larval densities [89,92,93], therefore, the variability in Dvv life history parameters associated with soil insecticide environments may be caused by the complex interaction of Dvv density, sublethal exposure/insecticide dose, and environmental conditions.

Liquid formulations of some carbamate, organophosphate, pyrethroid, and phenylpyrazole insecticides applied at planting or during the Dvv larval period have provided an alternative to granular formulations. Many liquid formulations can be applied in-furrow in starter fertilizer at planting [31,94] or chemigated through center pivot irrigation systems to control Dvv larvae [95]. In 1996, the Food Quality Protection Act altered the regulation of pesticides in the U.S. and the USEPA canceled uses of a number of organophosphate and carbamate insecticides, which has greatly reduced the insecticide options available for larval Dvv control.

### 2.2. Seed Treatments

The neonicotinoid insecticides thiamethoxam and clothianidin were initially registered as maize seed treatments in the early 2000s in the U.S. [96,97,98]. These seed treatments were primarily marketed at low rates (0.25–0.5 mg/seed) to control seedling maize insect pests and were quickly adopted by the seed industry. Today, most field-maize seed planted in the U.S. is treated with a low-rate neonicotinoid insecticide [39,99]. A higher rate (1.25 mg/seed) of thiamethoxam or clothianidin has been marketed for Dvv control. Clothianidin is very toxic to Dvv neonate larvae and some natural variability in susceptibility occurs among Dvv populations [100]. Field trial data consistently show that the 1.25 mg/seed neonicotinoid rate provides effective maize root protection when Dvv densities are low to moderate but protection is reduced under high Dvv larval densities [101,102]. To date there have not been any reports of field-evolved Dvv resistance to neonicotinoids deployed as seed treatments [102]. The future of neonicotinoids as seed treatments is unclear as environmental and nontarget concerns [103,104,105,106] have led to neonicotinoid bans in the European Union, restrictions in Canada, and a formal review of use in the U.S. [107,108,109]. Questions have also been raised as to whether an insecticidal seed treatment is necessary in all field situations because of the sporadic nature of many seedling pests [110]. Therefore, there is a need for greater understanding of actual pest pressure applied by the seedling pest complex in various crops/regions, methodology to assess the risk posed by seedling pests, and where appropriate, alternative technologies or strategies that can effectively protect seeds and seedlings from arthropods [110,111].

### 2.3. Foliar Insecticides

The application of foliar insecticides to suppress adult Dvv densities is used primarily for two purposes: (1) Protect maize during the pollination period from excessive adult Dvv silk feeding, which can interfere with pollination and result in poorly filled ears, and/or (2) reduce Dvv female density and associated oviposition to reduce potential larval injury the following season in continuous maize [112,113]. Initial experiments conducted by Hill et al. (1948) [23] demonstrated that DDT provided excellent control of adult Dvv. DDT was a key compound used to reduce Dvv silk feeding during the 1950s in Nebraska [25]. During the 1960s–1970s after field-evolved Dvv cyclodiene resistance limited larval control options, there was increasing interest in using adult foliar insecticide applications to reduce adult densities and oviposition in continuous maize fields to prevent the need for a soil insecticide the following year. Organophosphate and carbamate insecticides replaced DDT and provided control of adult Dvv [32,34,114]. A constraint of many products was low residual activity in the field because adequate residual activity coupled with proper application timing was needed to effectively manage rootworm densities [32]. Eventually, formulations of carbaryl (Sevin 4-Oil^®^, used mainly during the 1970s) and encapsulated methyl parathion (Penncap-M^®^, used primarily during 1980s–1990s) were developed that provided up to three weeks residual activity and became products of choice in many Dvv adult management programs [115]. The viable aerial application industry and the growing number of professional crop consultants led producers in some areas of south-central Nebraska to exclusively use adult control to manage Dvv in continuous maize [32,112,114].

The adult management strategy worked well in the western U.S. Corn Belt from the 1960s to early 1990s but reports of adult control failures increased during the 1990s in south-central Nebraska [115,116,117,118]. In areas where intensive use of Penncap-M^®^ was common, both rates of application and the number of applications per season increased as a result of reduced product efficacy [115]. Topical bioassays were used to document initial 16- and 9-fold field-evolved Dvv resistance to methyl parathion and carbaryl, respectively in 1995 [115], and vial bioassays were developed to monitor changes in geographical distribution of Dvv resistance in Nebraska over time [117,119,120]. Initially, Dvv resistance centered around two distinct focal areas separated by about 145 km in south central NE; Phelps/Kearney Counties and York County [117]. Dvv populations collected in counties between the focal areas were highly susceptible to methyl parathion suggesting that resistance may have evolved independently in each area. From 1996 to 2001, resistance intensity increased and areas between the initial focal areas previously identified as susceptible became highly resistant expanding the distribution of resistant Dvv populations throughout the Platte River Valley [121]. The use of adult control as a stand-alone Dvv management strategy gradually was abandoned during the late 1990s–2000s in Nebraska.

Research on the chemical ecology of *Diabrotica* species during the 1980s identified a variety of *Diabrotica* semiochemicals that provided new opportunities to manage adult Dvv populations with greatly reduced rates of insecticide. Semiochemicals included a variety of plant-derived attractants [122,123,124] and cucurbitacins (tetracyclic triterpenoids found in many cucurbits), which are arrestants and feeding stimulants for adult *Diabrotica* species [125,126,127]. A bait-type concept was developed in which semiochemicals were used to facilitate adult Dvv location and feeding on formulations that contain minute amounts of insecticide [128,129,130,131]. Initial formulations were granular and carbaryl was often used as the insecticide because of its efficacy as a Dvv oral poison. Efficacy of granular formulations was shown to be greatly affected by bait location and vertical distribution of adult Dvv in the plant canopy. In field maize, semiochemical-based baits were very effective up in the canopy but ineffective when placed on the ground [132,133]. To circumvent this problem, industry developed sprayable formulations that would adhere and dry on the plant versus granules that would roll off of plant leaves. MicroFlo Company (Lakeland FL) was issued a registration in 1992 for a sprayable microsphere formulation (SLAM) that contained cucurbitacin (*Cucurbita foetidissima* HBK root powder) and carbaryl [134]. Broadcast aerial applications of SLAM often provided >90% reduction of adult Dvv 24 h post application using only 10–13% of the active ingredient normally applied with conventional carbaryl applications [135,136]. Other companies subsequently developed adjuvant products that contained either *C. foetidissima* root powder (COMPEL: Scentry Inc. Billings, MT, USA; Cidetrak: Trece, Salinas, CA, USA) or bitter Hawksbury watermelon (*Citrullus vulgaris* Schrad.) juice (Invite: Florida Food Products, Eustis, FL, USA) that were marketed for tank mixing with very low rates of insecticide [134,137].

Pruess et al. (1974) [34] demonstrated in the late 1960s that application of ULV malathion over a 41.4 sq km area could reduce adult Dvv densities so larval densities the following year would be greatly reduced and not cause economic loss. This concept was revisited in a USDA-sponsored pilot areawide management program that was conducted at four 41.4 sq km locations in the Corn Belt from 1996 to 2002 to evaluate the efficacy of the semiochemical bait SLAM to manage Dvv population densities and crop injury [138,139]. The program clearly demonstrated that annual use of SLAM could effectively reduce adult Dvv and subsequent larval feeding injury the following season comparable to the level of control obtained with soil insecticides. Population management of Dvv was variable across sites as annual immigration of adults into the managed area prevented long-term suppression of Dvv over time. However, at some sites, the number of fields requiring treatment was significantly reduced over years in the managed area versus control companion area. The semiochemical bait approach significantly reduced the amount of ai applied by up to 20-fold and did not significantly affect densities of nontarget insects [139,140]. Previous research had shown that cucurbitacin was actually a repellent/antifeedant to some nontarget insects so nontargets could avoid the discrete bait droplets and not consume the toxin [141].

Part of the Dvv areawide management program was a project to monitor potential shifts in susceptibility of Dvv populations during the life of the pilot program. Annual collections of adult Dvv from managed and control companion areas of each program location were bioassayed with a diagnostic concentration of carbaryl (LC99). Significant reduction in Dvv susceptibility to carbaryl was detected in three of the four managed areas and a significant reduction in the responsiveness of Dvv to cucurbitacin was also observed at the same three sites [142]. Zhu et al. (2001) [143] conducted a separate monitoring study at the Kansas location and also reported rapid changes in susceptibility to carbaryl in the managed area after selection with SLAM over years. A similar shift in susceptibility was not observed in the companion control area. These results collectively suggest that areawide programs have the potential to select for resistance and that a strategy for managing and reducing selection pressure (e.g., rotation of insecticide ai) should be implemented. When the toxicity of various insecticide semiochemical bait mixtures was evaluated comparing Dvv organophosphate resistant and susceptible populations, results indicated that bait efficacy may be compromised by previously identified resistance and by insecticides that antagonize the feeding stimulation of the cucurbitacin in the bait [144]. Therefore, careful selection of insecticide active ingredient should be made if rotation of insecticides in baits over time is a resistance management goal.

The semiochemical bait concept or Dvv areawide management were not widely adopted in the U.S. Corn Belt. A number of factors may have contributed to this. Only small companies with limited research budgets and sales/marketing personnel developed the commercialized bait products. Larger agrochemical companies were committed to development of transgenic plants and companion seed treatment technologies as future Dvv management tools during the 1990’s–2000’s. Adult management is more knowledge and labor intensive than using a soil insecticide or planting a transgenic hybrid. In Nebraska, professional consultants are often hired to scout fields and properly time insecticide applications. Areawide programs involve a lot of organizational complexity and cooperation to uniformly apply specific management tactics over defined areas [145]. Therefore, farmers are required to relinquish some freedom to make independent insect management decisions on their farms. The end of the Dvv areawide pilot program coincided with the introduction of Dvv-active transgenic plant technologies and complementary neonicotinoid seed treatments that were quickly adopted [39,41]. The ability of farmers to incorporate a new technology with current practices and the perceived complexity of the innovation often impacts adoption [146]. The commercialization of transgenic plants simplified insect control by eliminating the need for soil-applied insecticides and provided excellent larval control [147]. While the semiochemical-bait concept still has merit, only a small niche market developed, and most bait or adjuvant products have been removed by industry from the market.

Since 2003, aerial or chemigated application of foliar-applied insecticides to manage arthropod pests is still commonly practiced in the western U.S. Corn Belt. However, planting of hybrids expressing Dvv-active Bacillus thuringiensis Berliner (Bt) proteins are often the centerpiece of Dvv management programs with fewer growers relying exclusively on insecticides to manage Dvv. Stand-alone adult Dvv management programs used in the 1960s–1990s have been replaced by periodic use of foliar applications to manage Dvv densities in continuous maize as a complement to other management tactics [148]. Regulatory reviews by the USEPA of older registered chemistries gradually removed many insecticides that were labelled for adult Dvv control leaving only pyrethroids and a few organophosphates as primary options. In the continuous maize system, the pyrethroid bifenthrin and organophosphate dimethoate are routinely used to manage other arthropod pests (i.e., western bean cutworm *Striacosta albicosta* (Smith), two-spotted spider mite *Tetranychus urticae* Koch, and banks grass mite *Oligonychus pratensis* (Banks) as well as Dvv, therefore, Dvv can receive selection pressure as a target or nontarget insect over time [148,149].

Results of active ingredient lab bioassays revealed that adult Dvv were very susceptible to bifenthrin during the 1990s in south-central Nebraska and southwestern Kansas [38,115]. However, from 2002–2014 there was a 40% increase in the use of bifenthrin in Nebraska [150]. From 2010–2012, anecdotal reports of reduced Dvv control with bifenthrin in parts of Nebraska and Kansas became more common. Follow-up active ingredient lab adult bioassays of Dvv field collections made in 2013–2014 from the affected areas of Kansas and Nebraska detected emerging Dvv field-evolved resistance to bifenthrin [149,151]. Initial resistance ratios were relatively low for adults (<10-fold), but subsequent active ingredient lab bioassays conducted with 2016 collections from the same Nebraska county as 2013–2014 collections revealed bifenthrin resistance ratios in the 4 to 55-fold range depending on the susceptible reference population used [148]. Dimethoate active ingredient lab bioassays with the same 2016-collected populations revealed a low level of Dvv resistance as well (RR: 3 to 16-fold) [148]. Indoxacarb, a recently registered insecticide labeled for Dvv adult control, was toxic to all adult Dvv populations bioassayed [148]. The bifenthrin resistance detected in the active ingredient lab bioassays was confirmed with commercial formulated bifenthrin using a simulated aerial application assay while the low level of dimethoate resistance detected in active ingredient bioassays was not apparent in formulated product assays [148]. This Dvv resistance problem is restricted primarily to southwestern areas of Nebraska and Kansas (and possibly northeastern Colorado).

Bifenthrin diagnostic concentration (LC99) bioassays of adult Dvv indicated that all populations tested from Corn Belt states east of Nebraska were very susceptible with increased survival in northeast NE and highest survival in southwestern areas of Kansas and Nebraska [149]. Dvv cross-resistance with the pyrethroids tefluthrin and cyfluthrin was also documented in lab bioassays [86,149]. Tefluthrin and bifenthrin are commonly used Dvv soil insecticides often applied in-furrow at planting throughout the Corn Belt while extensive use of aerial application of bifenthrin is limited to the western Corn Belt. Because resistance evolution attributed to direct selection of larvae has not been documented with soil insecticides placed in-furrow or over the row, the significant difference in Dvv susceptibility to bifenthrin between southwestern areas of Kansas and Nebraska versus geographic areas east of Nebraska strongly supports the working hypothesis that selection of adults is the main driver of observed Dvv pyrethroid resistance.

The Dvv organophosphate/carbamate resistance during the 1990s and Dvv pyrethroid resistance during the 2010s are the only known cases where the efficacy of Dvv soil insecticide active ingredients in bioassays or formulated product applied in-furrow or banded over the row was reduced by Dvv resistance [86,152]. In each case, resistance evolution was tied to selection of Dvv adults with foliar applied formulations leading to reduced efficacy of one or more soil-applied insecticides targeting larvae. Adult selection with the organophosphate methyl parathion reduced larval control efficacy with methyl parathion (not used as a soil insecticide), tefluthrin, and carbofuran [152]. The more recent pyrethroid selection of adults led to significant reductions in larval control efficacy with bifenthrin, tefluthrin, and cyfluthrin [86].

## 3. Mechanisms of Dvv Resistance to Insecticides

### 3.1. Organochlorides

The first investigations on mechanisms of Dvv cyclodiene resistance were mostly focused on comparative susceptibility levels and metabolic responses in resistant individuals. Experiments performed with field collected Dvv populations in the mid-1970s showed high levels of resistance to aldrin and heptachlor but not to other organochlorides such as DDT or methoxychlor indicating that the major selection for resistance in this species had resulted from cyclodiene soil treatments and suggested different metabolic pathways and/or modes of action between some organochloride insecticides [35]. An early investigation of in vivo insecticide detoxification mechanisms in Dvv showed that relative to organophosphates and carbamates, aldrin was very resistant to total detoxification but that it was readily converted to the more stable epoxide dieldrin [153]. Further examinations revealed a highly active cytochrome P450-dependent aldrin epoxidase system in cyclodiene resistant Dvv [154], which could not clearly explain the resistance phenotype since the epoxide dieldrin is equal to or more toxic to Dvv than the parental compound [155]. Another assessment showed that the susceptibility of Dvv to aldrin was increased by 9-fold when adults were maintained on diets other than corn suggesting that host-dependent alterations in aldrin metabolizing enzymes could contribute to the resistance mechanism [56]. Then a laboratory study demonstrated that aldrin resistant Dvv adults displayed a much higher conversion of epoxide dieldrin into aldrin trans-diol in the central nervous system relative to the whole body and also displayed cross-resistance to picrotoxinin [156], a compound thought to share a similar target site with cyclodiene insecticides [157,158].

Cyclodienes mode of action involves antagonism of the γ-aminobutyric acid (GABA) receptor in the nervous system thereby suppressing inhibitory post synaptic graded potentials [159,160]. The GABA receptors are ligand-gated chloride channels that regulate inhibitory potentials by controlling the flux of chloride ions through nerve cell membranes, and are important targets for several insecticides [161,162,163,164]. Target site mutations in the GABA-receptor (*Rdl*) were first detected in a dieldrin resistant strain of the fruit fly *Drosophila melanogaster* Meigen [165] and then in several other insect species [163,166]. In most cases studied, cyclodiene resistance was caused by a conserved point mutation which results in an amino acid substitution of an alanine either to serine or glycine within the second transmembrane domain (M2) of the GABA-receptor [163,166,167]. Investigations on cyclodiene-resistant Dvv populations collected throughout the U.S. Corn Belt from 2006 to 2011 confirmed a non-synonymous single nucleotide polymorphism (SNP) G/T at the GABA receptor cDNA position 838, which resulted in the alanine to serine change [168]. Results collected from Wang et al. (2013) [168] also suggested that a phenotypic gradient of increasing resistance levels from west to east in the U.S. Corn Belt historically documented [53,87] was correlated with higher frequencies of the resistance-conferring allele in the eastern-most populations.

For a period of nine years (1952–1961), the extensive use of aldrin in Nebraska provided high selection pressure over local Dvv populations and left considerable residue of this persistent organochloride and its epoxide, dieldrin, in the soil [47]. Data collected in Nebraska from 1962 through 1981 suggested a possible correlation between LD50 values for Dvv to aldrin and amounts of aldrin (<0.01 ppm) and dieldrin (0.02 ppm) remaining in the soils [47]. However, similarly high levels of aldrin resistance were still detected in the early 2000s in field-collected Dvv adults [87] in areas where soil samples contained residues as low as 0.002 ppm for aldrin and 0.006 ppm for dieldrin [169], which is likely below an effective concentration that would cause mortality even in susceptible populations. Additionally, Parimi et al. (2006) [87] found high levels of aldrin resistance in field collected Dvv populations that had been laboratory reared without insecticide exposure for 7–8 generations. Models of insecticide resistance evolution often share the central assumption that resistance is associated with a fitness cost because it involves a significant modification of a common phenotype, resistance genes generally have a low initial frequency, and resistance is rarely fixed in natural populations in the absence of selection [170,171,172]. Despite evidences for fitness cost associated with cyclodiene resistance in some insect species [173,174], cyclodiene resistant populations of *Drosophila* spp., *Blattella germanica* (L), and *Anopheles gambiae* Giles illustrate that *Rdl* mutations in the GABA-receptor can persist in the absence of selection [175,176,177]. The relatively high frequencies of *Rdl* mutations persisting in Dvv populations [168] is remarkable and may indicate lack of fitness cost associated with the resistance trait in this species [87].

### 3.2. Carbamates and Organophosphates

Carbamates and organophosphates are neurotoxins that bind to and inhibit acetylcholinesterases and prevent these enzymes from hydrolyzing the excitatory neurotransmitter, acetylcholine, thereby prolonging neuroexcitation of postsynaptic receptors in the nervous system [178,179]. Although both insecticide classes are acetylcholinesterase inhibitors, the pharmacokinetics of these compounds are not the same. The majority of organophosphate insecticides used for Dvv control contain the P = S moiety (e.g., parathion) and are metabolically activated by cytochrome P450 monooxygenases into more toxic true phosphates (e.g., paraoxon), which forms very stable bonds with serine residues of acetylcholinesterases [180]. The process of acetylcholinesterase recovery from organophosphate binding i.e., dephosphorylation, is very slow and mortality occurs before significant recovery [180,181]. Carbamates are direct inhibitors of acetylcholinesterases and metabolic activation is not required [180]. Moreover, the rate of acetylcholinesterase decarbamylation is significantly faster than dephosphorylation such that carbamates are generally less effective inhibitors of acetylcholinesterase [181]. Carbamates and organophosphates are both generally apolar compounds with ester bonds and can be converted in vivo into more water soluble and less toxic metabolites by hydrolytic esterases [182,183]. Additionally, several P450-dependent oxidative processes are involved in detoxification of these insecticides [184].

The most common mechanism of carbamate and organophosphate resistance in insects is through enhanced insecticide detoxification [184], but mutations in acetylcholinesterases that minimize insecticide binding have also been documented [185,186]. Mechanisms involved in carbamate and organophosphate resistance in Nebraska Dvv populations exhibiting high levels of resistance to methyl parathion and carbaryl have been extensively studied. Cross-resistance has been identified not only to other organophosphates and carbamates, but also to pyrethroids, which were not used extensively in affected areas, suggesting that common metabolic pathways shared between these different insecticide classes were involved in the resistance mechanism [115,152,187]. Additional in vivo and in vitro experiments confirmed significative differences in oxidative (i.e., P450-based) and hydrolytic (i.e., esterase-based) insecticide metabolism in the resistant Dvv populations [119,152,187,188] and provided collective evidence for a number of different attributes of the resistant populations.

Dvv populations with similar levels of resistance to methyl parathion and carbaryl in insecticide bioassays [115] displayed variable isoforms and activity levels of both P450s and esterases suggesting that resistance mechanisms were different among the populations examined [187]. Enhanced esterase activity detected in resistant Dvv populations was involved in more efficient detoxification of both methyl parathion and carbaryl [187,188]. P450-dependent bioactivation of methyl parathion into the more toxic metabolite methyl paraoxon was consistently reduced among resistant populations and in combination with enhanced esterase activity conferred high resistance levels [187]. Cytochrome P450-dependent N-demethylation was enhanced in at least one resistant Dvv population and was a primary metabolic event involved in carbaryl detoxification [188]. Both methyl parathion and carbaryl resistance that was selected in Dvv adults conferred resistance to larvae but did not result in equal cross-resistance to soil-applied O-ethyl-substituted organophosphates (terbufos and chlorpyrifos) and carbamates (carbofuran) [152]. Enhanced esterase activity was the most consistent resistance mechanism identified among both adult and larval stages, and cross-resistance with pyrethroids was higher in Dvv larvae (tefluthrin) than in adults (bifenthrin) [115,152]. The complexity of these results indicates that it is probably inappropriate to generalize a common Dvv resistance mechanism although common attributes of enhanced metabolic detoxification were detected.

Further investigations of the organophosphate- and carbamate-resistant Dvv identified through Northern blotting experiments revealed higher expression of CYP4 P450 genes in Dvv populations that were significantly resistant to methyl parathion and carbaryl [189]. In addition, three distinct esterase isozyme groups were identified in Dvv and the activity of a specific group of esterase isozymes (group II) was consistently higher in all resistant Dvv populations [190]. The activities of these isozymes based on native gel electrophoresis were identified as a reliable marker for detection of resistance [120]. Purification and characterization of group II esterases demonstrated that they were present in all Dvv populations, but were overproduced in resistant phenotypes indicating quantitative genetic differences rather than qualitative changes in physical chemical properties of these enzymes [191]. Reciprocal crosses of resistant Dvv populations then revealed that inheritance of methyl parathion resistance was not exclusively correlated with inheritance of elevated esterase activity [192] confirming earlier findings that additional factors other than just enhanced hydrolytic metabolism were involved in the resistance, such as differential P450-mediated oxidation [187,188,189]. Similar to cyclodiene resistance, Dvv resistance levels to methyl parathion remained relatively stable over many years in the absence of selection in both laboratory and field populations suggesting lack of fitness cost associated with the resistance trait and persistence of correspondent resistance alleles [87]. However, it is unclear whether there is a lack of fitness cost or if the univoltine life cycle prevented/delayed the fitness cost to manifest.

### 3.3. Pyrethroids

The pyrethroid insecticide class includes neurotoxins that bind and disrupt voltage-gated sodium channels associated with axonal transmission [193,194,195], although voltage-gated calcium and chloride channels have also been identified as secondary targets [196,197,198,199,200,201]. Once pyrethroids bind to sodium channels, axons experience a higher influx of sodium ions, prolonged sodium inactivation and repetitive electrical discharges that disrupt the normal flow of information through the central nervous system [202]. Pyrethroids are generally divided into two classes based on the symptomology, and by the presence or absence of an α-cyano group in the molecular structure. Type I compounds (e.g., permethrin, tefluthrin, bifenthrin) are characterized by causing restlessness, incoordination, and prostration, whereas Type II (e.g., deltamethrin, λ-cyhalothrin, β-cyfluthrin) contain an α-cyano group in the molecule structure and cause incoordination, convulsions, and intense hyperactivity [200,203,204]. Although this distinction among the two subclasses exists, it is possible to find some pyrethroids exhibiting intermediate properties [203,204,205,206].

Multiple studies in different insect species have shown that enhanced activity of some cytochrome P450s, glutathione *S*-transferases and esterases can provide more efficient pyrethroid detoxification in resistant insects, while sodium channel mutations (*kdr*) can affect the binding of these insecticides to the target site [184,207,208,209]. Lab bioassays have demonstrated that Dvv resistance to bifenthrin is partially suppressed by inhibitors of esterases and P450s, and that bifenthrin resistant Dvv populations are cross-resistant to a soil applied pyrethroid (tefluthrin) and to the organochloride DDT [151]. DDT was banned and no longer used in the U.S. since the early 1970s, but it targets the voltage gated sodium channels similarly to pyrethroids making it useful to diagnose potential target site mutations in cross-resistance lab bioassays [210,211,212]. Interestingly, Dvv resistance to DDT has not been previously reported. The initial screening for mechanisms of Dvv pyrethroid resistance suggested that both enhanced metabolism and sodium channel mutations could be involved in the resistance trait [151].

Further investigations revealed that bifenthrin- and tefluthrin-resistant Dvv populations also showed resistance to a Type II pyrethroid (cyfluthrin), reduced susceptibility to the organophosphate dimethoate, and increased susceptibility to the oxadiazine indoxacarb [86,148]. Pyrethroids, carbamates, and organophosphates all possess ester bonds and can be detoxified by the same hydrolytic enzymes [182,183]. Conversely, indoxacarb becomes more toxic through esterase/amidase hydrolytic bioactivation [213,214,215]. Therefore, reduced efficacy of the organophosphate dimethoate and negative cross-resistance with indoxacarb observed in pyrethroid-resistant Dvv populations [148] suggested enhanced hydrolytic enzyme activity as has been reported in other insect species [216,217,218,219,220]. A biochemical evaluation of total protein collected from Dvv adults confirmed significantly higher activity of esterases and P450s in pyrethroid-resistant Dvv populations [221] supporting previous work that suggested the involvement of these enzyme groups in the resistance trait [148,151].

Laboratory selection of insecticide resistant populations is often used to examine the genetic basis of resistance, such as mechanisms, heritability, and time for resistance development [222,223,224]. Laboratory selection of a pyrethroid-resistant Dvv population demonstrated a major genetic contribution to the resistant phenotype and suggested that pyrethroid resistance evolution may occur rapidly in this species (~7 generations) under continuous adult selection [221]. Toxicological and biochemical comparisons between lab-selected and field-derived pyrethroid resistant individuals suggested that variable esterase and P450 isoforms/activity may be selected across Dvv populations showing similar levels of resistance [148,221]. RNA-seq gene expression analysis confirmed major overexpression of several CYP6 P450s in pyrethroid resistant Dvv populations, possible contribution of proteins with nervous system functions, and that enhanced activity of esterases previously detected could be a qualitative difference rather than quantitative [225]. However, *kdr* sodium channel mutations were not detected in pyrethroid resistant Dvv populations suggesting that pyrethroid resistance and DDT cross-resistance observed in Dvv populations is not associated with target site insensitivity [221,225].

## 4. Conclusions

The history of insecticide use to manage Dvv clearly reveals a common thread among all documented cases of Dvv resistance evolution, namely population management. The broadcast cyclodiene soil applications and the organophosphate, carbamate, and pyrethroid foliar applications all exposed a large part of the target Dvv population to some level of toxicant without any structured refuge. This combined with repeated use of the same insecticides over Dvv generations led to field-evolved resistance. The structure of agricultural enterprises in the western Corn Belt (large irrigated monocultures, continuous maize, strong aerial application and livestock industries) has led to a tradition of extensive foliar insecticide use over time to manage relatively high annual densities of Dvv and other secondary insect pests. This has contributed to the sequential occurrence of Dvv resistance evolution to different active ingredients not observed in other parts of the U.S. Corn Belt. The history of Dvv resistance to insecticides illustrates the contrasting selection intensity that insecticide application strategies may provide to different life stages of insect pests and shows that insecticide resistance may evolve quickly in field populations if adequate insecticide resistance management practices are not adopted. Dvv cross-resistance to different insecticide classes and long persistence of insecticide resistance mechanisms even in the absence of selection exemplifies how levels of resistance estimated in the laboratory can have variable practical implications on the field efficacy of formulated insecticides.

In the transgenic era, foliar insecticides will still be used as a tool to prevent excessive Dvv silk-clipping when needed, especially in seed production fields. The stand-alone adult management programs are no longer recommended partly because many longer residual insecticide products have been removed from the market and the adult emergence and oviposition periods associated with transgenic seed blends (“refuge in the bag”) are extended after sublethal exposure to Cry toxins in transgenic plants [226,227,228,229]. This makes it difficult to obtain the required level of oviposition suppression to prevent economic injury the following season without applying multiple adult insecticide applications. A key message from the Dvv insecticide use story presented in this review is that more holistic Dvv management strategies are needed, which combine and rotate multiple tactics at the farm level. Foliar and soil insecticides still have a place as complementary tactics with crop rotation, conventional and transgenic hybrids, and potentially biological control (e.g., nematodes [230,231]) if used within an integrated pest management framework to manage annual densities/crop injury and resistance over time [41,232]. History has clearly documented that Dvv is highly adaptable to selection pressure from management tactics [52,115,149,233,234,235] so the probability of selecting for field-evolved resistance to any future Dvv control technology will be high if it is repeatedly used as a stand-alone management tactic.

## Data Availability

Data sharing is not applicable to this article as new data were not created or analyzed.
